# Inter-familial and intra-familial phenotypic variability in three Sicilian families with Anderson-Fabry disease

**DOI:** 10.18632/oncotarget.18250

**Published:** 2017-05-29

**Authors:** Antonino Tuttolomondo, Irene Simonetta, Giovanni Duro, Rosaria Pecoraro, Salvatore Miceli, Paolo Colomba, Carmela Zizzo, Antonia Nucera, Mario Daidone, Tiziana Di Chiara, Rosario Scaglione, Vittoriano Della Corte, Francesca Corpora, Danai Vogiatzis, Antonio Pinto

**Affiliations:** ^1^ U.O.C di Medicina Interna con Stroke Care, Dipartimento Biomedico di Medicina Interna e Specialistica (Di.Bi.M.I.S), University of Palermo, Palermo, Italy; ^2^ CNR-IBIM: Institute of Biomedicine and Molecular Immunology “A. Monroy” Palermo, Palermo, Italy; ^3^ Stroke Unit, Neurology, Saint Andrea Hospital, La Spezia, Italy; ^4^ Department of Clinical Neurological Sciences, Western University, London, Ontario, Canada

**Keywords:** Anderson-Fabry disease (AFD), family, variability

## Abstract

**Background:**

Anderson-Fabry disease (AFD) is an inborn lysosomal enzymopathy resulting from the deficient or absent activity of the lysosomal exogalactohydrolase, α-galactosidase A. This deficiency, results in the altered metabolism of glycosphingolipids which leads to their accumulation in lysosomes, thus to cellular and vascular dysfunction. To date, numerous mutations (according to recent data more than 1000 mutations) have been reported in the GLA intronic and exonic mutations. Traditionally, clinical manifestations are more severe in affected hemizygous males than in females. Nevertheless, recent studies have described severe organ dysfunction in women.

**The aim of the study:**

This study reports clinical, biochemical, and molecular findings of the members of three Sicilian families. The clinical history of these patients highlights a remarkable interfamilial and intrafamilial phenotypic variability which characterizes Fabry disease relative to target organs and severity of clinical manifestations.

**Discussion:**

Our findings, in agreement with previous data, report a little genotype-phenotype correlation for the disease, suggesting that the wide phenotypic variability of Anderson-Fabry disease is not completely ascribable to different gene mutations but other factors and mechanisms seem to be involved in the pathogenesis and clinical expression of the disease. Moreover, this study emphasies the importance of pedigree analysis in the family of each proband for identifying other possibly affected relatives.

## INTRODUCTION

Anderson-Fabry(AFD) disease, also known as Fabry disease, is a rare X-linked lysosomal storage disorder characterized by an altered glycosphingolipid metabolism because of a mutation in GLA gene encoding α-galactosidase A [1–4]. This enzymatic defect results in pathological accumulation of glycolipids, such as globotriaosylceramide (Gb3), in lysosomes of vascular endothelium and several cell types causing multi-organ involvement [5].

The classical phenotype of Fabry disease typically occurs in males in childhood or adolescence; it consists of gastrointestinal symptoms as diarrhoea, abdominal pain, fever, hypohidrosis, acroparesthesias, angiokeratomas, *cornea verticillata.* Over the years, affected subjects develop, progressive renal disease, cardiac and cerebrovascular complications leading to premature death in the fourth and fifth decades of life [6].

Some patients present later onset form of the disease, often exhibiting a single organ dysfunction.

Approximately 70% of carrier women exhibit several symptoms of Fabry disease, even though their clinical manifestations are usually later in onset and milder than in affected males [7, 8]. However, some females may also present severe complications relative to its effect on men. The moderate and mild phenotypes and the wide variability which characterizes Fabry in females are partly explained by the process of *lyonization* or X-chromosome inactivation which occurs randomly. Because of random X-chromosome inactivation, enzymatic GLA assay may provide enzymatic levels within the normal range; thus, different from male patients, genetic testing is the only method which allows the detection of female carriers. Consequently, molecular analysis should be performed in women who are suspected to suffer from Fabry disease; moreover it is essential for a conclusive correct genetic counselling [9].

Previously, the estimated prevalence of Fabry disease was approximately one out of 40000 to one out of 117000 in male subjects [10]. These data have been recently reviewed; in fact, newborn screening programs suggest an unexpectedly elevated rate of GLA mutations causing the disease [11]. Moreover, the diffuse employment of genetic testing among patients with mild, late-onset clinical manifestations or doubtful symptomatology has allowed detecting numerous novel mutations over the past few years [12]. The role of intronic mutations and polymorphisms of a single nucleotide are yet to be well defined. However, they recently appear to contribute to the extreme clinical variability and severity of the disease.

Numerous studies have demonstrated the molecular heterogeneity of Fabry disease. To date, researchers have identified more than one thousand mutations, a large part of them are private mutations that are to say specific to a particular family.

The study of several families reveals that despite a single mutation, the clinical expression of the disease may be significantly heterogeneous, as reported in the renal involvement of a large Slovenian family [13].

A considerable variability exists in phenotypic expression of the disease and organ involvement, suggesting that in addition to genotype, other factors may contribute and influence the clinical expression of this lysosomal disorder, such as environmental factors, polymorphisms of other genes (interleukin 6, nitric oxide synthase), modifiers of lysosomal pH, intra-gene factors, and epigenetics. In the light of these and other unknown phenomena, the correlation between genotype and phenotype is not so close thus the result is unreliable [14].

**Figure 1:**
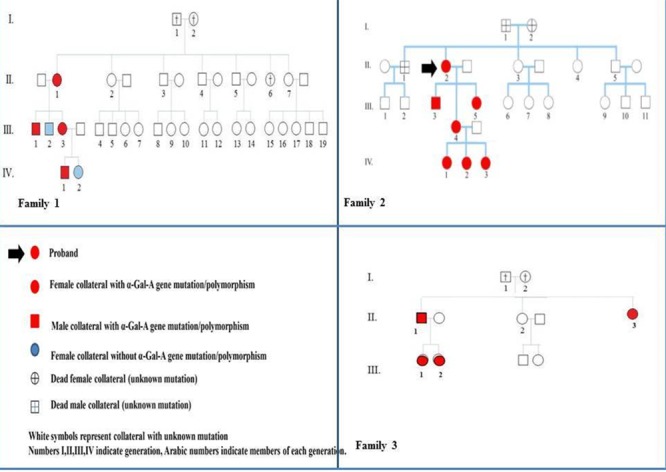
Family trees and pedigree symbols of three sicilian families with Anderson-Fabry disease

We report the clinical, biochemical, molecular study of three Sicilian families (see Figure 1 and Table 1); our results confirm the wide phenotypic variability of Anderson-Fabry disease among members of a given family with a specific gene mutation and possibly may evoke further debates about this interesting topic.

**Table 1:** Plasma α-galactosidase A activity, GLA mutation, clinical manifestations in three Sicilian families

Organ involvement and symptoms											
	Age/ sex	α-Gal	GLA mutation	Skin	Eye	Pain	Gastrointestinal	Ear	Heart	Brain	Kidney
**Pedigree 1**											
	**59/F**	**3.7**	**-10 C>T; IVS2-76_80del5; IVS4-16 A>G; IVS6-22 C>T** **(Heterozygote)**	**−**	**−**	**+**	**−**	**+**	**−**	**+**	**−**
	**30/M**	**1**	**-10 C>T; IVS2-76_80del5; IVS4-16 A>G; IVS6-22 C>T** **(Hemizygote)**	**−**	**−**	**+**	**+**	**−**	**−**	**−**	**+**
	**41/F**	**4.1**	**-10 C>T; IVS2-76_80del5; IVS4-16 A>G; IVS6-22 C>T** **(Heterozygote)**	**−**	**−**	**+**	**−**	**+**	**−**	**−**	**−**
	**9/M**	**1.65**	**-10 C>T; IVS2-76_80del5; IVS4-16 A>G; IVS6-22 C>T** **(Hemizygote)**	**−**	**−**	**+**	**+**	**−**	**−**	**−**	**−**
**Pedigree 2**											
	**58/F**	**3.3**	**c.614delC** **(Heterozygote)**	**−**	**−**	**+**	**−**	**−**	**+**	**+**	**−**
	**28/M**	**0**	**c.614delC** **(Heterozygote)**	**+**	**+**	**+++**	**−**	**−**	**+**	**−**	**+**
	**36/F**	**3.4**	**c.547G>A p.G183S** **(Hemizygote)**	**+**	**−**	**++**	**++**	**−**	**−**	**−**	**++**
	**34/F**	**3.3**	**c.547G>A p.G183S** **(Heterozygote)**	**−**	**−**	**+**	**−**	**−**	**−**	**−**	**+**
**Pedigree 3**											
	**55/M**	**0.4**	**c.547G>A p.G183S** **(Hemizygote)**	**−**	**−**	**−**	**−**	**+++**	**+++**	**−**	**++**
	**18/F**	**3**	**c.547G>A p.G183S** **(Heterozygote)**	**−**	**−**	**++**	**−**	**−**	**−**	**−**	**++**
	**20/F**	**3.6**	**c.547G>A p.G183S (Heterozygote)**	**−**	**−**	**−**	**−**	**−**	**−**	**−**	**−**
	**58/F**	**3.7**	**c.547G>A p.G183S (Heterozygote)**	**−**	**−**	**−**	**−**	**−**	**−**	**−**	**+++**

+, mild; ++ moderate; +++, severe

## RESULTS

After obtaining informed consents, we performed the clinical evaluation, enzyme assay for measuring GLA activity, genetic testing in 12 members of three Sicilian families.

### Family 1: polymorphism -10 C>T; IVS2-76_80del5; IVS4-16 A>G; IVS6-22 C>T

In this family, the proband (case II/1), a 59-year-old woman, presented cerebrovascular manifestations of Fabry disease. She had occasional detection at brain MRI of extensive, symmetric white matter hyperintensities (WMHLs) with relative sparing of subcortical white matter. MRI was performed because she often complained of a diffuse headache associated with tinnitus and dizziness After administration of mini-mental state examination (MMSE) she did not show any objective evidence of cognitive impairment. She also suffered from early-onset hypoacusis and acroparesthesias, since the age of 35. In the light of brain MRI findings, the absence of cerebrovascular risk factors, after the exclusion of autoimmune, thrombophilic disorders Anderson-Fabry disease was suspected. Her alpha-galactosidase A activity was 3.7 nmol/ml/h, which is compatible with a heterozygote female subject or healthy subject.

The proband’s son (CASE III/1), a 30-year-old male, has suffered from acroparesthesias and hyperhidrosis since adolescence. During childhood, he complained gastrointestinal symptoms (recurrent nausea, abdominal pain and diarrhoea). A mild impaired renal function has been detected at the age of 29 years old (GFR=65 ml/min). Plasma α-Gal A activity was significantly decreased, a common typical value for hemizygous men with Anderson-Fabry disease.

Another member of this family group, a 41-year-old female (case III/3), suffered from a paucisymptomatic variant of AFD characterised by acroparesthesias, hyperhidrosis, exercise and heat intolerance. She has reported episodic painful crisis characterized by burning sensation primarily in the extremities and radiating inwards to the limbs, for ten years. However, magnetic resonance imaging of her spinal cord showed multiple cervical herniations that could in part clarify her neuropathy. Also, she presented bilateral hypoacusis and central vestibular abnormalities of probable vascular origin. No neuroimaging findings are suggestive of cerebral involvement from Fabry disease. A-Gal A activity was 4.1 nmol/ml/h. To date, enzymatic replacement therapy has not been started yet.

The proband’s grandson (case IV/1), a 10-year-old child, presented a classic phenotype of AFD with early onset of symptoms. He has mainly suffered from gastrointestinal involvement (chronic recurrent abdominal pain) and peripheral neuropathy since the age of 4. Gastrointestinal involvement consisted of recurrent episodes of diffuse abdominal pain primarily in the meso-hypogastric region, postprandial bloating, early satiety and later, chronic diarrhoea, trouble gaining weight. Also, he presented typical *Fabry crises.* Plasma enzyme activity was typically decreased (1.65 nmol/ml/h). He started enzymatic replacement treatment at the age of six years.

Molecular genetics revealed the same polymorphism for each component member of this family: -10 C>T; IVS2-76_80del5; IVS4-16 A>G; IVS6-22 C>T [15].

### Family 2: mutation c.614delC

The proband, a 57-year-old female, has complained mainly of central and peripheral neurological involvement with recurrent ischemic strokes, since the age of 45; and acroparesthesias, sensitive polyneuropathy since the age of 30. Her brothers (case II/1 and case II/5), presented respectively an ischemic stroke at the age of 40 and a cardiac death for myocardial infarction. Her mother underwent dialysis for end-stage renal disease. Moreover, the proband suffered from cardiovascular disease (coronary artery disease, valvulopathy, peripheral vasculopathy) in the absence of neither cardiovascular risk factors nor autoimmune, thrombophilic disorders.

Her son (case III/3), a 29-year-old man has developed the classic phenotype of Fabry disease. He had typical features of AFD with systemic organ involvement consisting of small-fiber peripheral neuropathy, autonomic dysfunction, skin lesions (multiple angiokeratomas), ocular manifestations (cornea verticillata), mild renal impairment (GFR: 71 ml/min), cardiac involvement (moderate left ventricular hypertrophy). The patient had the lowest plasma alpha-galactosidase A activity among our patients (0 nmol/h/ml) (15).

In this family, other two members (case III/4 and case III/5) had mild-moderate clinical manifestations. The first was a 36-year-old female who has complained gastrointestinal symptoms, as cramp-like abdominal pain and diarrhoea since adolescence. Also, she presented angiokeratomas affecting the genital region, acroparesthesias, diffuse arthralgias. Finally, kidney involvement was detected, urinary protein excretion was 250 mg/24h. After this additional renal finding, ERT was started. Her sister, 37 years (case III/5) had a late onset milder form of the disease characterised by acroparesthesias and later mild proteinuria. [data not yet published].

Genetic DNA sequence analysis detected a point mutation c.614delC [15].

Molecular genetics of other three young members of the family 2 (cases IV/1, case IV/2, case IV/3) did not reveal any mutation in detecting the polymorphism: -12G>A;IVS4+68 A>G; IVS6-22C>T.

### Family 3: mutation c.547G>A

This family consisted of 4 members suffered from Fabry disease, with the same mutation responsible for clinical manifestations.

The proband (case II/1), a 55-year-old man was a patient with the cardiac variant of Anderson-Fabry disease. He had concentric left ventricular hypertrophy (LVH), associated with thickening of the mitral valve and a shortened PR interval. Echocardiographic study showed a mild reduction of ejection fraction. Early symptoms were angina on effort and exertional dyspnea. His exercise stress test was positive for myocardial ischemia but coronary angiography ruled out significant stenosis. In addition, an extracardiac hallmark of AFD was a chronic renal disease (stage 3). His younger sister (case II/2) had hypertrophic cardiomyopathy. His older sister (case II/3) was affected by end stage renal disease; cardiac evaluation did not show signs of cardiovascular involvement.

The proband’s youngest daughter (case III/1), a 17-year-old adolescent, presented with proteinuria (the first measurement of daily urinary protein excretion was 250 mg/24h) and mildly reduced kidney function (GFR: 73 ml/min, CKD-EPI). The only extra-renal features were acroparesthesias since the age of 12. The concentration of the biomarker lyso-Gb3 was increased to 7,5 ng/ml (reference ≤1,7 ng/ml); she has undergone ERT for two years. Case III/2 a 20-year-old female did not complain of symptoms and/or signs which may be suggestive of Fabry disease.

The mutation was detected in exon 3 of the GLA gene: c.547G>A.

### Enzyme activity

In family 1, the two hemizygous subjects (case III/1, case IV/1) had a decrease in plasma, α-galactosidase A activity to 1 nmol/ml/h and 1,65 nmol/ml/ml, respectively. Enzyme activity was normal in the heterozygous female subjects: the proband’s plasma enzymatic activity was 3,7 nmol/ml/h, case III/3 showed a normal value of 4,1 nmol/ml/h.

In family 2, plasma α-galactosidase A activity was significantly reduced in subject III-3, effectively its value was equal to 0 nmol/ml/h. Enzyme activity was within the normal range in proband II-2, subject III-5, subject III-4 who were heterozygous (>3 nmol/ml/h).

The hemizygous patient of family 3 had a relevant alpha galactosidase A deficiency in plasma 0,4 nmol/ml/h, compatible with the affected male with Fabry disease. The heterozygous subjects showed normal values of alpha-galactosidase A activity.

Analysis of lyso-GB3 concentrations was performed in some patients as case III/1 of family 3. She had increased levels of Lyso-GB3 (7,8 ng/ml, reference <1,8 ng/ml).

### Genetic analysis

In this study, 4 hemizygotes, including 1 proband and 3 newly detected hemizygotes, and 8 heterozygotes were diagnosed by gene analysis.

Family 1 showed these sequence alterations: −10 C N T; IVS2-76_80del5; IVS4-16 A N G; IVS6-22 C>T, corresponding to genetic polymorphism identified by sequencing of the GLA gene. This genetic polymorphism has been detected in Fabry patients according to literature data. Recent studies have described this intronic haplotype in asymptomatic and paucisymptomatic variants of Fabry disease and in patients with small fibre neuropathy of unknown etiology [18, 19].

However, to date, no conclusive data exist regarding the pathogenic role of this complex intronic haplotype in Fabry disease.

Family 2 presented a point mutation, exactly a deletion of a single nucleotide (cytosine) in exon 4 of the GLA gene causing a frameshift mutation: c.614delC. Only the 29 year-old subject was hemizygous, whereas heterozygosity was detected in the proband a 57 year-old woman, and in two female subjects aged 36 and 38, respectively.

The third family showed a deletion in exon 3 of the GLA gene (c.547G>A) resulting in a glycine to serine substitution at codon 183 (p.G183S). This mutation has previously been described as disease causing by Shabber et al [20]. The proband was the only hemizygous of this family. Heterozygosity was reported in 4 subjects of this group.

## DISCUSSION

Fabry disease is characterized by an evident phenotypic variability. A considerable variability exists in the severity of disease and organ complications even between members of the same family; however the relationship between clinical manifestations, biochemical abnormalities, genetic mutations has not been clearly established. Some authors such as Vedder have not well defined a correlation between plasma and urinary Gb3 concentrations and most of the clinical signs and symptoms such as microalbuminuria, pain, and hearing loss [25].

Some specific mutations seem to be related to certain phenotypes with a predominantly single organ involvement, both cardiac variant, and cerebrovascular variant have been described. These forms are usually associated with a late onset phenotype [26, 27, 28].

Our results show a wide interfamilial and intrafamilial variability in the phenotypic expression of AFD, as regards both the target organs and disease severity. The great heterogeneity in disease expression is stressed by differences in most of the symptoms in family members carrying the same mutations.

Plasma α-galactosidas A activity and gene analyses were performed in all 13 members of three families that have come to our attention; four hemizygous and nine heterozygous were recognised.

Family 1, consisting of 4 members, is associated with polymorphism *-10 C>T; IVS2-76_80del5; IVS4-16 A>G; IVS6-22 C>T* that was detected in AFD patients according to literature source. The significance of this haplotype in Fabry pathogenesis is still unknown, however, it is likely suggestive of AFD. In this first pedigree, a decrease in enzymatic activity was observed in two male patients; instead, the other members’ values were within the normal range [20, 23, 24].

The hemizygous subject and 3 heterozygotes from the second pedigree had a deletion of a single nucleotide in exon 4 leading to a frameshift mutation [21]. The proband and her two daughters had normal levels of plasma α-Gal A activity, whereas the hemizygous patient’s enzyme activity was found to be 0 nmol/ml/h.

Finally, family 3 consisted of 5 members (one man and four women, aged 18 to 59 years) with a mutation in exon 3 causing a Gly183Ser substitution [22]. The proband had a decrease in α-Gal A activity, whereas 4 heterozygous female subjects had normal values.

Gene analysis is crucial for an accurate diagnosis because plasma alpha-galactosidase A activity was normal in most of the heterozygotes. Also, clinical and instrumental evaluation of patients is important for a more accurate diagnostic assessment. This consideration is relevant especially now that enzyme replacement treatment is available for this metabolic disorder that was incurable until about fifteen years ago [29].

All hemizygous patients among the three families, except the proband of family 3, who suffers from a typical cardiac variant of the disease, suffered from small fiber neuropathy, hypohidrosis, heat/exercise intolerance, gastrointestinal symptoms and two of them presented a classical phenotype of the disease ((case IV/1 in family 1 and case III/3 in family 2). With regards to cardiac variant, it should be noted that epidemiological studies have reported a prevalence of Fabry cardiomyopathy from 1 to 12% among middle-aged patients with unexplained cardiac hypertrophy, thus it is advisable to screen these subjects for α-galactosidase A deficiency, and equally, patients suffering from known Fabry disease should be screened at an early stage for cardiac involvement, since treatment may not be effective once cardiac fibrosis has occurred [30].

Middle aged women of our studied families experienced predominantly cerebrovascular complications of AFD, whereas younger females have acroparesthesias that they have complained since adolescence, heat or cold intolerance, persistent non-specific gastrointestinal symptoms, and incipient kidney injury as manifested by new-onset proteinuria; the latter has justified the initiation of enzyme replacement treatment for three of them (case III/4, case III/5 of family 2, case III/1 of family 3). Only one heterozygous woman (case III/4 of family 2) had skin lesions (angiokeratomas).(see Table 1).

In each family, there was no correlation between the type of mutation found in α-GAL A gene and Fabry phenotype.

Medical history of these families, just as other pedigrees, highlights a wide phenotypic variability characterising Fabry disease in terms of organ involvement and disease severity, showing how a given mutation could express several phenotypes. It is well-known that Fabry disease is characterized by a poor phenotype-genotype correlation, thus a given mutation in a family may be responsible for different phenotypes.

Classically affected males experience first symptoms in infancy or adolescence (most of them complain of hypohidrosis, heat and cold intolerance, gastrointestinal symptoms, angiokeratoma) and have a more severe manifestation of the disease than heterozygous women, that are often asymptomatic according to *Lyon*'s hypothesis of random inactivation of the X Chromosome [31] which partially explains less severe manifestations and wider variability in women. Moreover, literature reports many patients with only late involvement of one or two organs; these variants are usually associated with mutations that cause limited residual enzyme activity [4, 14].

On the basis of the current pathogenic hypothesis, it may be difficult to explain the lack of symptoms in patients with low enzyme activity. In this regard, Cammarata et al. [3], reported the case of two male members of the same family that showed the exonic mutation M51I; although their enzymatic activity was zero, they presented different clinical phenotypes. The eldest was asymptomatic, the youngest showed severe clinical manifestations; the current pathogenetic hypothesis does not clarify the poor symptomatology in the eldest hemizygous patient with absent enzyme activity. It is likely that the penetrance of different mutations is influenced by other variables (genetic, epigenetic or environmental factors) [4].

Recent studies report that heterozygous females may not be only mere carriers but women may experience serious clinical manifestations and irreversible organ damage. The extreme phenotypic variability does not seem entirely attributable to the numerous described mutations of GLA gene. Some authors have described mutations that were detected in patients with mild form and later also identified in severe cases [32].

Bono et al. have recently described mild or asymptomatic variants with residual enzymatic activity. The authors identified polymorphisms in the promoter region of the GLA gene in 12% of their analyzed subjects, and of these, 99% showed several simultaneous polymorphisms throughout the entire sequence; in particular they detected simultaneously two polymorphisms the -10 c>t, IVS2-76_80 del5, IVS4-16 A>G, IVS6-22C>T, which is identical to the polymorphism identified in our family 1, and -12 g>a, IVS4+68 A>G, IVS6-22C>T polymorphisms in 8.9% and 3.7% of the subjects; however the significance of this haplotype in the pathogenesis of Fabry disease remains still unknown. Also, other authors identified the first haplotype in patients with small fibre neuropathy of unknown etiology [23, 24].

Such examples and others suggest the existence of additional factors that impact on the phenotype of the disease (I.e.modifier genes, enviromental factors). In this regard, some hypotheses have been suggested: as the presence of residual enzyme activity in patients with classic form [4]; different levels of enzyme synthesis; different catalytic activity of the enzyme; the influence of environmental factors and not yet well-known genetic factors. To date, this appears an interesting and suggestive issue to continue to analyse [33]. Also, epigenetic mechanisms of gene regulation influence the penetrance of different disease-causing mutations. The latter hypothesis suggests that chromatin alterations of specific single nucleotide polymorphisms represent a platform that allows them to influence the phenotype. Chromatin variation is associated with distinct single nucleotide polymorphisms suggesting that the variation may serve as a platform to enable these SNPs often found in non-coding regions of DNA to influence phenotype; no study to our knowledge has yet assessed the role of these mechanisms in SNP associated with Fabry disease or other lysosomal disorders [14, 34–38].

Spence et al [38]. showed that there was a considerable variation in the clinical expression of the disease suggesting the presence of other interacting factors that may influence phenotype. Hamers et al [39]. suggested that clinical variability may be caused by other genes and/or environmental factors. A previous study by Beyer et al reported that two brothers with Fabry disease presented differences in severity of cardiovascular and renal symptoms [39–41].

Lukas et al [36]. have highlighted and reaffirmed that to date a decrease in enzyme activity is the most relevant factor influencing the phenotype and its severity. However, the importance of modifier genes and epigenetic factor on clinical manifestations requires further researches to be confirmed.

Verovnik et al. have reported a significant variability in organ involvement in a Slovenian family with Fabry disease highlighting how a single specific mutation (N272S) may be responsible for heterogeneous deterioration of renal function; despite this mutation caused classical Fabry disease they described a wide variability of phenotypes among the seven hemizygotes of that family in particular, relative to renal disease [13].

No study has currently clarified whether the above mentioned processes individually or together explain the wide phenotypic variability and the different organ damage associated with Fabry disease and other lysosomal storage diseases. The role of intronic mutations and single nucleotide polymorphisms still has to be clarified. It has been shown that aberrant splicing of the messenger is the cause of at least 15% of human genetic diseases, highlighting the importance of studying intronic regions of the gene, too [42]. Since non-coding regions are not routinely evaluated, the occurrence of intronic-disease causing alterations should induce to analyse them when sequencing the GLA gene and to perform in these subjects further clinical and instrumental evaluation.

It is desirable that the GLA gene sequencing should be performed including routinely non-coding regions so as to identify possible disease-causing intronic lesions; thus, subjects that are affected from these polymorphisms should be undergone clinical and instrumental evaluation.

Patient follow-up will help us to better understand the role of intronic mutations on clinical manifestations of the disease. It is essential to establish a diagnostic process which is based on suggestive symptomatology and the presence of a classical GLA mutation, but this consideration is not possible for all phenotypes and genotypes of a patient with suspected Fabry disease thus in this circumstance a strict, careful clinical evaluation is required before starting ERT. For subjects with intronic mutations or single type polymorphism, avoiding in principle enzyme replacement treatment should not be recommended because it may lead to problems with the eventual progression of the disease especially in males but event in women and milder variants [15].

It is relevant the knowledge of the mechanism by which a mutation causes an altered function of the enzyme because numerous mutations are potential targets for alternative therapeutic strategy different from enzymatic replacement treatment, as pharmacological chaperone therapy [12].

Our results and other numerous case studies display a noteworthy intrafamilial and interfamilial clinical variability in terms of organ involvement and sometimes in terms of severity of the disease.

Our findings support previous observations highlighting the difficulties encountered by physicians in making a correct prognosis especially for young patients in reference to their family history.

In view of this, it becomes important the identification of biomarkers in order to establish the severity of disease and to monitor the effect of substitute treatment. To date, no markers are available in order to detect patients with increased risk of early complications. It is thus important recognising early signs and symptoms, bioumoral alterations so it would be possible to start early enzyme replacement therapy.

However, lyso Gb3 has been assuming a considerable role since it has been reported that its levels correlate with the severity and progression of the disease and they seem to be modified by enzyme replacement therapy [43, 44].

Further studies will clarify the pathogenesis of Fabry variability in order to improve the diagnostic and prognostic accuracy in Fabry disease. In addition, the study of large families may allow the identification of interacting factors influencing clinical expression and that may acquire a relevant value in order to decide the best time to start treatment.

## MATERIALS AND METHODS

Measurement of enzyme activity: alpha-galactosidase A, activity was measured by Dried Blood Filter Paper (DBFP) in all family members.

Refrigerated K-EDTA whole blood samples, arrived at our laboratory (max 24 hrs) from different hospitals via a prepaid shipping agency (TNT). Samples were listed and spotted for biochemical and genetic analysis. Microdisk (6 mm diameter) was prepared from 9cm Whatman No. 113 fast flow/coarse filter disk paper by using a common hole punched for documents. Whole blood of 5.5 μl is dispensed onto microdisk, using a Drummond positive pressure pipette. The disks were dried for two hours covered with a brief easy membrane (sigma MW08253). Ten disks of the same sample were put down in a horizontal line of the dark multiwell plate, with each letter on the microplate identifying a different sample. Samples were analysed in duplicate against a blank containing the spot and, in order, 250 μl of ethylendiammine 0.1 M pH 11.4 and 70 μl of assay mix (below). To assess the specificity of the enzymatic reaction 3 μl of alphagalactosidase A, inhibitor (deoxygalactonojirimycin-hydrochloride (Sigma D9641)) was added onto two DBFP (these samples were incubated for 1 hr with assay mix). Finally, 70 μl of assay mix was added to each well with a multichannel pipette. The dark plate was covered with Breathe-easy membrane and incubated in a rotating shaker (37°C—300 rpm). The kinetics of the reaction was measured at intervals of 1 hr, 2 hrs and 3 hrs and was stopped by using 250 μl of ethylendiammine 0.1 M pH 11.4. The kinetics assay was carried out in a 384-well plate format using 1 nM enzyme with varying concentrations of substrate. Initially, 10 μl/well, of the varying concentrations of the substrate was added to the plate. The reaction was initiated by the addition of 20 μl/well of enzyme solution. The 4MU-α -Gala stock solution was serially diluted 1:1.5 to give eight concentrations. The final concentrations of the substrate used in the assay were 500, 333, 222, 148, 98.8, 65.8, 43.9, and 29.3 μmol/L. Stop solution of 30 μl/well was added after 2, 4, 6, 8, 10, and 12 minute incubation times at RT. A quantitative research graph of the free fluorophore, 4-methylumbelliferone (4MU), in the same volume of assay buffer and stop solution was generated for calculating the enzyme product. The plate was read in the ViewLux plate reader at an emission wave-length of 440 nm and an excitation wave-length of 365 nm. In order to determine the type of inhibition and the inhibition constant of lansoprazole, five enzyme kinetics plots were generated in the presence of 80, 40, 20, 10, and 0 μmol/L of the inhibitor.

### Reaction mix

Reaction mix was prepared in citrate, phosphate buffer (CPB) 0.15 M pH 4.5 (P4809-50 TAB Sigma, a tab dissolved in 33 mL H2O to obtain the wanted molarity). 4-Methylumbelliferyla-D-galactopyranoside 5mM (Sigma M763) strained by 0.45nm syringe filter, and N-acetyl-Dgalactosamine (Sigma A2795) 0.25 M, were mixed in a ratio of 2.5:1. Seventy microliters of this mixture was used for each well.

### α-Galactosidase A inhibitor A

2 mg/mL of deoxygalactonojirimycin-hydrochloride (Sigma D9641) was prepared in distilled water. Prepared solution was then diluted in a ratio of 1:3 with CPB (citrate phosphate buffer). Using 3 μl of the prepared dilution, the final concentration in the assay was 1.3 × 10−7 M [16–18].

### Alpha-galactosidase A activity

The values of enzyme activity obtained with Dried Blood Filter Paper (DBFP) range from 0 to 14 nmol/h/mL. In brief, a 3.2-mm disk was punched and incubated at pH 4.4 and at 37°C with 4-methyl-umbelliferyl-alpha-galactopyranoside as a substrate and N-acetyl-D-galactosamine as an inhibitor for alpha-galactosidase B. Enzymatic activities measured on a Thermo Life Science fluorometer (Thermo Electron Corporation, Waltham, MA, USA) were expressed as nanomoles of substrate hydrolyzed per mL of blood per hour. Reference values for alpha-galactosidase activity are: males: N or =1.2 nmol/mL/h; females: N or =2.8 nmol/mL/h [19]. Typical values for h hemizygous Fabry patients range from zero to 1.5 nmol/hr/mL, while, healthy female and male subjects range from 3 to 14 nmol/h/ml).

Gene analysis: Direct sequencing of α-GAL A gene was conducted in all family members. DNA was extracted from whole blood. Peripheral blood was collected, using EDTA as an anticoagulant, from patients with clinical manifestations related to FD symptomatology. Genetic analysis was performed by high resolution melting (HRM) analysis on DNA samples, isolated from whole blood, using the Light Cycler 480 system. PCR products presenting melting curves in different positions or shapes from those of the wild-type control were sequenced to identify the suspected mutations.

### Additional molecular genetics

To demonstrate intronic alterations, we applied the mismatch discover method known as HRM (high resolution melting) to identify human single nucleotide polymorphisms or mutations in GLA gene. For nuclease digestion, we performed one step of hybridization between wt and target DNA. Nuclease digestion required the addition of specific mismatch restriction enzyme called surveyor nuclease. Finally, polyacrylamide gel analysis was performed as protocols. We used high resolution melting (HRM) analysis for the rapid and sensitive detection of mutations in clinical samples. HRM assays were performed on the LightCycler® 480 Real-Time PCR System (Roche Diagnostics), analysed with the software Gene-Scanning Software Version 1.2 (Roche Diagnostics). CEL I and ENDO-1 endonucleases were used and primer sets were designed to cover the 7 exons and most common intronic mutation (10 C N T; IVS2- 76_80del5; IVS4-16 A N G; IVS6-22 C N TVS4 + 919G N A) of GLA gene.

The concentration of the biomarker lyso-Gb3 in dried blood spot was measured using HPLC and tandem mass spectrometry.
